# Comparison of Ultrasound Descriptors of Abnormally Invasive Placenta (AIP) over the Course of the Second and Third Trimester—Is an Increase Verifiable?

**DOI:** 10.3390/jcm10214960

**Published:** 2021-10-26

**Authors:** Monika E. Gorczyca, Stephanie Springer, Petra Pateisky, Johannes Ott, Barbara Ulm, Kinga Chalubinski

**Affiliations:** Department of Obstetrics and Gynecology, Medical University of Vienna, 1090 Vienna, Austria; monika.gorczyca@meduniwien.ac.at (M.E.G.); stephanie.springer@meduniwien.ac.at (S.S.); petra.pateisky@meduniwien.ac.at (P.P.); johannes.ott@meduniwien.ac.at (J.O.); barbara.ulm@meduniwien.ac.at (B.U.)

**Keywords:** abnormally invasive placentation (AIP), placenta accreta spectrum (PAS), placenta, ultrasound, percreta, accreta, increta, preterm birth, hemorrhage, progress, aggravation

## Abstract

Limited data exist regarding the course of abnormally invasive placentation (AIP) (=placenta accreta spectrum (PAS)) during the 2nd and 3rd trimester, although this knowledge would be important for optimal patient care. In this retrospective single-center longitudinal cohort study, potential aggravation of AIP was evaluated in 37 patients with ultrasound (US) pictures stored on a minimum of two visits. Five raters, blinded to diagnosis and gestational age, judged the degree of AIP as recommended by the International Society for PAS. The probability of invasiveness was estimated as absent, low, intermediate, severe (0–3 points), the extent as absent, focal, diffuse (0–2 points), and the presence and appearance of each US-sign as absent, mild, severe (0–3 points). None of the 10 judged signs appeared more severe (*p* ≥ 0.41) with progressing pregnancy. Neither the number of positively scored US-signs (earlier scan; 6.14 ± 2.06, later scan; 5.94 ± 2.16; *p* = 0.28), nor the estimated probability & extent of AIP rose (3.69 ± 1.15 vs. 3.67 ± 1.22; *p* = 1.0). Test-retest reliability corroborated excellent agreement between visits (mean number of positive US-signs ICC (3,1) = 0.94, 95% CI 0.91–0.97; *p* < 0.0001). Overall, there was no clinically detectable increase in invasiveness over the course of the 2nd and 3rd trimester. This should be further evaluated in prospective studies.

## 1. Introduction

Abnormally invasive placentation (AIP), also called placenta accreta spectrum (PAS), is one of the most morbid conditions obstetricians may encounter. Following the trend of increasing cesarean delivery, the incidence of AIP has risen steadily in recent years [1,2]. Despite its growing rates, AIP remains a rare condition (0.79–3.11 per 1000 births after prior cesarean) [3] and most obstetricians have personally managed only a small number of cases. AIP leads to failure of the placenta to separate normally from the uterine wall at delivery. This can result in massive hemorrhage causing high maternal morbidity and mortality [4]. Concomitantly, there is an also increased fetal morbidity and mortality, mainly due to preterm delivery [5]. Pregnancy following placenta accreta is at increased risk for adverse outcomes such as recurrent accreta, uterine rupture, and peripartum hysterectomy [6].

Diagnosis of AIP is complex, as there are different sonographic signs of invasion, which are also dependent on disease severity [7]. Researchers often refer to the same sign differently which makes comparison of studies to date difficult. To ensure that all operators are using the same description for the same sign, the International Society for Placenta Accreta Spectrum (IS-PAS, formerly known as International Society for Abnormally Invasive Placenta (IS-AIP)), recently introduced a standardized description and nomenclature for all currently known AIP ultrasound signs [8,9]. This enables comparable standards not only for future research but also for individual diagnosis.

There is still a lack of data regarding many issues in AIP management. Although prenatal diagnosis has been shown to improve the outcome of patients delivering in centers with high expertise, there is still debate on when to scan and refer women at risk, and on optimal delivery time-point [5,10]. Current consensus recommends delivery between weeks 34–37, to balance the risk of maternal hemorrhage in the setting of preterm labor, but also to prevent fetal morbidity due to prematurity [5,11].

However, the above mentioned uncertainties in standardized procedures are reflected in data of large population-based studies, where antenatal diagnosis was, surprisingly, also associated with delivery in earlier gestational weeks. This was partly attributable to emergency cesarean sections [12,13,14], but Fitzpatrick et al. report that actually 70% of their population were delivered electively in gestational weeks 27–36 [14], thus potentially risking unnecessary fetal morbidity [15].

Regarding those findings and our personal experience, one might suspect that the delivery time-point chosen by some caregivers is not only directed by guidelines, but sometimes by fear of hemorrhage or fear of disease progress. It is crucial to know the course of the disease for counselling affected patients and choosing the right time-point for diagnosis and further monitoring. Diagnosis of AIP is usually made in the 2nd trimester, but until now there are only limited data dealing with the course of AIP during 2nd and 3rd trimester.

Thus, the aim of our study was to survey whether the extent of placental invasion and the presence of ultrasound signs suggestive of AIP aggravates with advancing pregnancy. Therefore, we conducted a retrospective single-center longitudinal cohort study in which five raters, blinded to the patients’ diagnosis and gestational age, compared the degree of AIP in the 2nd and 3rd trimester.

To our knowledge, to date no study has been performed in which raters were not only blinded for degree of AIP but also for the timepoint of the sonography scan, thus, allowing for neutral evaluation of the placental invasiveness degree over the course of pregnancy.

## 2. Materials and Methods

In this retrospective single-center longitudinal cohort study, we compared the degree of AIP over the course of the 2nd and 3rd trimester. Since January 2001, at the Department of Feto-maternal Medicine of the Medical University of Vienna, Austria, sonographic screening for placental invasion has been established as a routine procedure for all pregnant women with one or more risk factors for AIP, i.e., placenta previa, history of more than one uterine surgery (Cesarean section, curettage, myomectomy) and/or a history of manual placenta separation during a previous delivery (according to the guidelines for peripartum hemorrhage of the German society of Gynecology and Obstetrics [16]). Ultrasound examinations for this placental invasion screening were performed using commercially available real-time equipment, with standard 3.75-MHz linear or sector transducers transabdominally, and 7.5-MHz sector transducers, trans-vaginally, grey scale and color Doppler pictures were obtained. Of all women delivering at our department between January 2001 and July 2017, 61 displayed AIP, confirmed perioperatively and/or histo-pathologically (as defined by the FIGO guidelines on diagnosis of PAS [17]). The medical history of each patient was evaluated and those with an adequate number of high resolution ultrasound pictures taken and stored at a minimum of two time-points during the 2nd and 3rd trimester, were included to the study. Thirty-seven patients with placenta accreta, increta or percreta were included. Patients without perioperative and/or histologic verification of AIP, with only one in-house US examination before delivery, or with an insufficient number of retrospectively assessable US scans were excluded. Five raters scored the ultrasound scans. The rater team consisted of one expert rater (a highly experienced ultrasound operator with >20 years’ experience in placental pathology US, a member of the IS-PAS), two ob-gyn residents, and two medical-technical assistants trained in prenatal ultrasound. All raters were blinded to the final diagnosis, the exact gestational age and trimester of the evaluated pictures. All patients’ pictures were presented in a random order. In addition, to ensure blinding, the raters were told that they were reviewing pictures of patients with either placenta previa only or with AIP. The staff member who presented the archived US scans on a screen to the raters was also blinded to the patients’ gestational age and diagnosis. Reporting of signs of invasiveness was performed according to the recommendations of the international consensus statement of the IS-PAS [8,9]. Each ultrasound sign was rated as either absent (0), mild (1), or severe (2), according to its presence and severity appearance. Further, all raters gave their individual estimate of probability of invasiveness, and scored it as absent (0), low (1), intermediate (2) or severe (3). The raters also estimated the extent of invasiveness as absent (0), focal (1) (minor or concerning only a small part of the placenta) or diffuse (2) (Table 1). The expert rater additionally screened the pictures for placental degenerations, i.e., irregular non-vascularized areas suggesting the presence of hematomas or placental infarction. The presence of degenerations served as the control variable, as their presence is not directly associated with placental invasiveness [18,19,20]. Statistical evaluation comprised the following aspects: comparison of severity of appearance of each individual ultrasound sign between an earlier and a later visit during the 2nd and 3rd trimester. The number of positively vs. negatively scored US-signs at both visits was evaluated. Additionally, a score summing-up the presence and severity of the appearance of the ultrasound signs per patient was calculated (“US-Score”, see Table 1). A second pooled score (“Probability-Score”) was calculated for the sum of the estimated AIP probability and the estimated extent. The calculated scores and the list of the evaluated US signs are presented in Table 1.

Statistical analysis was performed with SPSS 24. Numerical data are provided as mean and standard deviation, whereas categorical data are shown as a number (frequency). Testing for normality of distribution was performed by the Shapiro-Wilk-Test. Wilcoxon signed-rank test was used to compare the appearance of severity of the positively scored ultrasound parameters, the number of positively scored US signs, the “US-Score” and the “Probability-Score” over time. McNemar’s test was used to compare the presence of degenerations. Spearman correlation coefficient was used for assessing correlation between the “US-Score” and the “Probability-Score” with the final diagnosis. Comparison of correlation coefficient magnitude was performed with Cocor software [21]. To corroborate the consistency and reproducibility of the results, Intraclass Correlation Coefficient (ICC) was calculated [22,23]. ICC estimates and their 95% confidence intervals were calculated to assess test–re-test reliability, based on absolute agreement and the two-way-mixed-effects model (mean rating of all raters *k* = 5). Based on the 95% confidence interval of the ICC estimate, values less than 0.4, between 0.4–0.59, between 0.6–0.74, and greater than 0.75 are indicative of poor, fair, good, and excellent reliability, respectively [24]. As two methods can show excellent correlation despite the presence of significant systematic bias, to rule-out systematic scoring bias Bland-Altman analysis was additionally performed [25]. A *p* value below 0.05 was considered significant. To ensure sensitivity for even only slightly significant changes between the earlier and the later measurement, we purposefully did not use corrections for multiple testing.

## 3. Results

Mean maternal age was 33.8 yrs. (95% CI 32.2–35.5). Mean gestational age at delivery was 34.2 weeks, (95% CI 33.3–35.0). Mean number of previous pregnancies was 4 (min. 2, max. 10), mean parity was 3 (min. 0, max. 6). All patients had at least one risk factor for AIP [26]: history of previous cesarean section (27 patients), history of previous uterine surgery, including curettage (18 patients), history of IVF (nine patients), history of placental separation failure (nine patients), placenta praevia marginalis or totalis (34 patients).

Of the 37 patients included in the analysis, 10 (27.0%) had the final diagnosis placenta accreta, 8 (21.6%) placenta increta and 19 (51.4%) placenta percreta. Mean gestational age for visit 1 was 24.6 weeks. (95% CI 23.1–26.1.) and for visit 2 was 32.3 weeks. (95% CI 31.5–33.2). Maternal and gestational characteristics are given in Table 2.

**Figure 1:**
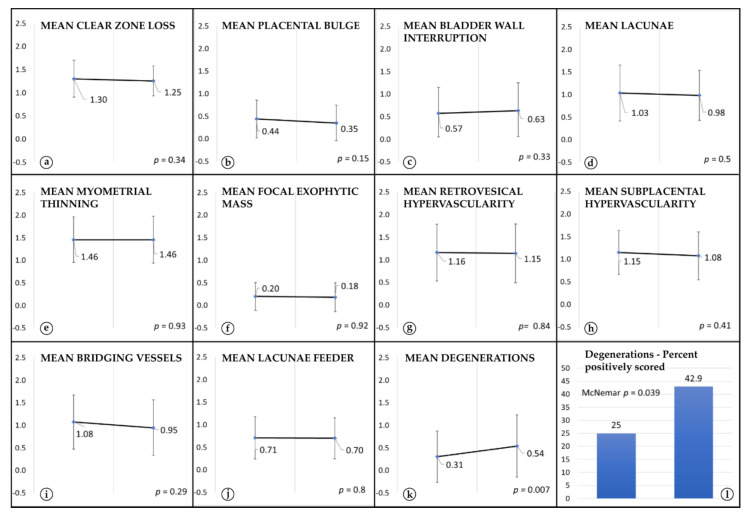
Comparison of severity estimates: There was no visible change in severity appearance of the 10 AIP ultrasound signs (**a**,**c**–**j**) except for a decrease in severity of the placental bulge (**b**) and an increase for the comparator sign, i.e., placental degenerations (**k**) (y-axis: mean severity of appearance ± std. dev. per visit). Significantly more degenerations could be noted at the later US scan (**l**, percent of positively scored).

In the case of 9 AIP signs (loss of clear zone, bladder wall interruption, lacunae, myometrial thinning, presence of focal exophytic mass, uterovesical hypervascularity, sub-placental hypervascularity and lacunae feeder vessels), there was no measurable change in severity of appearance between the earlier vs. the later US scan (*p* ≥ 0.05) (Figure 1). A difference was noted only for the placental bulge. Surprisingly, the mean severity decreased from a mean score of 0.44 to 0.35 (*p* = 0.015) (Figure 1). Example scans for three patients are depicted in Supplementary Materials, Figure S1. The severity of appearance and the amount of the control variable, i.e., placental degenerations, increased with progressing pregnancy and was significantly more severe and more often noted at the later US scan (mean severity of appearance 0.31 vs. 0.54; *p* = 0.007), present in 25% of the patients at the earlier scan vs. 42.9% patients at the later scan, McNemar test: *p* = 0.039. (Figure 1).

The number of positively scored US signs per patient did not increase with progressing pregnancy (earlier scan; 6.14 ± 2.06, later scan; 5.94 ± 2.16; *p* = 0.28) The compound “US-Score” (number of positively scored signs and their estimated severity) also did not differ (earlier scan; 8.51 ± 3.66, later scan; 8.41 ± 3.89; *p* = 0.79). The “US-Score” correlated significantly with the final diagnosis (earlier scan; ρ = 0.66, later scan ρ = 0.60; *p* < 0.001 for both). The mean number of positive signs and the mean “US-Score” as rated by each rater are depicted in Table 3.

There was no difference in the estimated extent and probability of AIP (“Probability-Score”; Table 2) between visits (earlier scan; 3.69 ± 1.15, later scan; 3.67 ± 1.22; *p* = 1.0). The “Probability-Score” correlated significantly with the final diagnosis regardless of timepoint (earlier scan; ρ = 0.74, later scan; ρ = 0.67; *p* < 0.0001 for both). Moreover, the magnitude of correlation did not differ between visits (z = 0.58; *p*= 0.57).

For the mean “US-Score” the ICC (3,1) was 0.95 (95% CI 0.92–0.97; *p* < 0.0001) and for the mean number of positive US Signs ICC (3,1) was 0.94 (95% CI 0.91–0.97; *p* < 0.0001), corroborating the results of the Wilcoxon Signed Rank Test and demonstrating excellent test-retest agreement.

**Figure 2:**
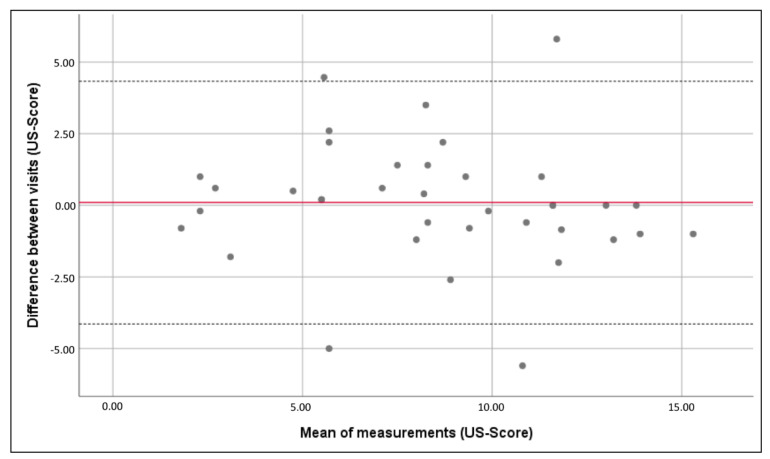
No visible trend in the distribution of the mean difference between visits (“US-Score”, dotted lines show 95% CI).

The Bland Altman plot further confirmed agreement between the US scans, showing that there was no difference in scoring distribution (mean difference in “US-Score” = 0.098, 95% CI 4.14–4.33, Figure 2).

## 4. Discussion

In some cases, sonographic features of AIP may be detected as early as in the 1st trimester, but there are limited data on their course during the 2nd and 3rd trimester, when diagnosis is usually made [27,28,29].

Although the incidence of AIP has risen progressively in recent decades, there are many uncertainties regarding care for AIP patients and standardization of optimal antepartum and intrapartum management is still ongoing [5]. There are no published, randomized, controlled trials demonstrating the superiority of a single management strategy. Thus, counselling, delivery-timepoint and pregnancy management is based mostly on retrospective case series and expert opinion, and for that reason has inevitably biases [5,30]. Notably, the depth of villous invasion at delivery is a major predictor of outcome. While patients with placenta accreta might be treated conservatively, patients with placenta increta or percreta often require caesarean hysterectomy. Therefore, it is important to know the course of the disease to ensure optimal care and counseling for each patient.

In the current study, we used a predefined set of ultrasound markers, which in previous studies was found to be of high utility in the diagnosis of AIP [8,9]. The main result of the present data set was that the rate of invasiveness did not progress significantly over the course of the 2nd and 3rd trimester.

In detail, we could not detect an increase in the number of positively scored US-signs (Table 2). Furthermore, none of the examined ultrasound signs of abnormally invasive placentation appeared significantly more severe when comparing the earlier scans with later US-scans (Figure 1). However, there was a slight decrease in the appearance severity of the placental bulge. This might be due to the fact that the lower uterine segment unfolds with time, and therefore a placental bulge might look less pronounced. To ensure transparency and to be sensitive for even only slightly significant changes, we purposefully did not use corrections for multiple testing. If we had applied a correction for multiple testing, such as Bonferroni-Holm, this would have changed the *p*-values for the comparison of the placental bulge from 0.015 to 0.15, rendering it also insignificant. 

In contrast, the presence of the comparator ultrasound sign, i.e., placental degenerations, increased significantly, which is in accordance with the fact that they are a sign of placental ageing (Figure 1) [18,19,20]. This underlines the finding that, while placental ageing and placental growth is ongoing, invasiveness does not progress after the end of the 2nd trimester.

In the hands of experienced operators, ultrasound has a sensitivity and specificity of up to over 90% for the diagnosis of AIP [31,32]. Nevertheless, interpretation of ultrasound pictures is to some extent subjective; while objectively two sonographers see the same picture, they might judge it differently according to their experience. The subjective estimate of disease probability and extent was included in the proposal of pro forma AIP reporting of the IS-PAS [8,9]. According to this recommendation, we also asked the raters for their personal estimate of disease probability and extent. The raters did not judge the placentae as more invasive when looking at later pregnancy weeks US-scans. The calculated “Probability Score” had a high and significant correlation with the final diagnosis regardless of gestational age (ρ = 0.74 & 0.67; *p* < 0.0002). There was no difference between the correlation magnitude and the final diagnosis (z = 0.58; *p* = 0.57). Test–retest reliability assessed by ICC corroborated excellent agreement between both visits. Bland Altman analysis ruled out systematic rating error (Figure 2).

Like many other studies on AIP, our study has the limitation of being conducted retrospectively on a relatively small number of patients, which is due to the rarity of the disease. Due to the retrospective design we had to exclude patients where no pictures of the uterovesical plane were stored at a minimum of two timepoints, thus probably missing patients with non-anteriorly lying placentae or less severe diagnosis.

To the best of our knowledge, to date, only two studies have compared the appearance of ultrasound signs over the course of pregnancy. Comstock et al. evaluated 14 patients and found that, in three, ultrasound signs, which were not present in the 2nd trimester, were visible in the 3rd. However, their study was not designed for comparison of invasiveness, but for a general evaluation of US-signs present in AIP [33]. In their retrospective study, Calì et al. found significant changes in ultrasound signs when comparing the 1st with the 3rd trimester, but not between the 2nd and 3rd trimester. The latter finding is in accordance with our results. Nonetheless, the authors concluded that they have had the personal experience that AIP is a progressive condition which needs to be monitored closely during the 3rd trimester to ascertain depth and topography of invasion [34]. It has been shown that, in some cases, early signs of severe AIP can be detected in the 1st trimester. Nonetheless, the final degree of invasiveness should be reached in the middle of the 2nd trimester, when the placentation process is completed [35]. Accordingly, in our study, we could not find measurable aggravation of invasiveness over the course of the 2nd and 3rd trimester. Thus, while looking at a patient’s US-scans over time, sometimes we might have the personal impression of a progress or change. However, according to our data and the results of Calì et al. [34], changes in appearance are, rather, attributable to placental ageing and not to clinically significant aggravation of invasiveness.

Most patients receive the diagnosis in the 2nd trimester. Therefore, if a patient’s diagnosis is placenta accreta in the 27th gestational week, this diagnosis will remain the same and will not change or aggravate into a placenta increta or percreta. Although heavy bleeding or preterm rupture of membranes might happen in patients with AIP, making earlier delivery necessary, for the majority, a delivery time-point between weeks 34 and 36 is optimal, thus avoiding unnecessary morbidity of the neonate. Considering the fact that the majority of women with a higher degree of invasive placenta will undergo hysterectomy, and will not be able to have another child, it should be one of the highest priorities of caregivers not only to avoid maternal hemorrhage, but also to avoid extreme preterm deliveries and the risks they pose to the health of the neonates [11,15].

We believe that knowledge about the course of the disease is important for doctors to ensure optimal counselling and surveillance during pregnancy. Prospective studies should be conducted to further examine this important topic.

## Figures and Tables

**Table 1:** Calculated scores and the list of evaluated US signs ^1^.

**Calculated Scores & Values**
“Severity of appearance”—mean estimate of severity for each US sign
“US-Score”—mean severity & number of positive signs per patient
Mean number of positively scored signs
“Probability-Score”—the reviewers subjective estimate of AIP probability & extent
**Ultrasound Signs for US-Score Calculation and Reporting of Positively Scored Signs**
Loss of clearzone
Myometrial thinning
Abnormal placental lacunae
Placental bulge
Focal exophytic mass
Uterovesical hypervascularity
Subplacental hypervascularity
Bridging vessels
Placental lacunae feeder vessels
**Estimates of AIP Probability & Extent**
Raters’ estimate of AIP probability
Raters’ estimate of AIP extent

^1^ According to Collins et al., 2016 [8].

**Table 2:** Maternal and gestational characteristics & final diagnosis.

Sample Size *n* = 37	Mean	95% CI for Mean	Median	Min	Max
Maternal age yrs	33.8	32.1–35.5	34.4	23.7	43.2
Delivery week	34.2	33.3–35.0	34.7	26.3	37.4
Delivery before week 37 + 0 n (%)		34 (91.9)			
Delivery before week 34 + 0 n (%)		11 (29.7)			
**Final diagnosis**					
placenta accreta n (%)		10 (27.0)			
placenta increta n (%)		8 (21.6)			
placenta percreta n (%)		19 (54.4)			
**Week of pregnancy**					
US-Scan 1	24.6	23.1–26.1	25.4	13.4	32.1
US-Scan 2	32.3	31.5–33.2	33.1	25.1	35.4

**Table 3:** Mean number of positive signs and the mean “US-Score” (number of positively scored signs and their estimated severity) as rated by each rater. Rater 1 represents the expert sonographer.

	Mean Ultrasound-Score					Mean Number of Positive Signs				
	US-Scan 1		US-Scan 2			US-Scan		US-Scan		
Rater	Mean	SD	Mean	SD	*p*	Mean	SD	Mean	SD	*p*
1	8.3	4.18	8.6	3.84	0.35	6.3	2.50	6.5	2.18	0.35
2	8.5	4.74	8.4	4.79	0.19	5.9	2.68	5.9	2.85	0.19
3	8.1	4.77	7.6	5.04	0.41	5.7	2.76	5.3	3.04	0.33
4	8.2	3.20	8.8	3.62	0.54	6.2	1.99	6.4	2.06	0.52
5	9.3	5.22	8.9	5.21	0.50	6.2	2.67	5.9	2.56	0.433
Mean of 5 Raters	8.5	3.66	8.4	3.89	0.79	6.1	2.06	5.9	2.16	0.28

## Data Availability

The data are available on request to the corresponding author.

## Supplementary Materials

The following are available online at https://www.mdpi.com/article/10.3390/jcm10214960/s1, Figure S1: Example ultrasound pictures of 3 AIP cases.
