# Supplementary material for: Comparison of Ultrasound Descriptors of Abnormally Invasive Placenta (AIP) over the Course of the Second and Third Trimester—Is an Increase Verifiable?
Source: J Clin Med. 2021 Oct 26;10(21):4960. doi: 10.3390/jcm10214960 (PMC8584941; doi:10.3390/jcm10214960)
Supplement: Supplementary file 1 [file jcm-10-04960-s001.zip › jcm-1379484-supplementary.pdf]

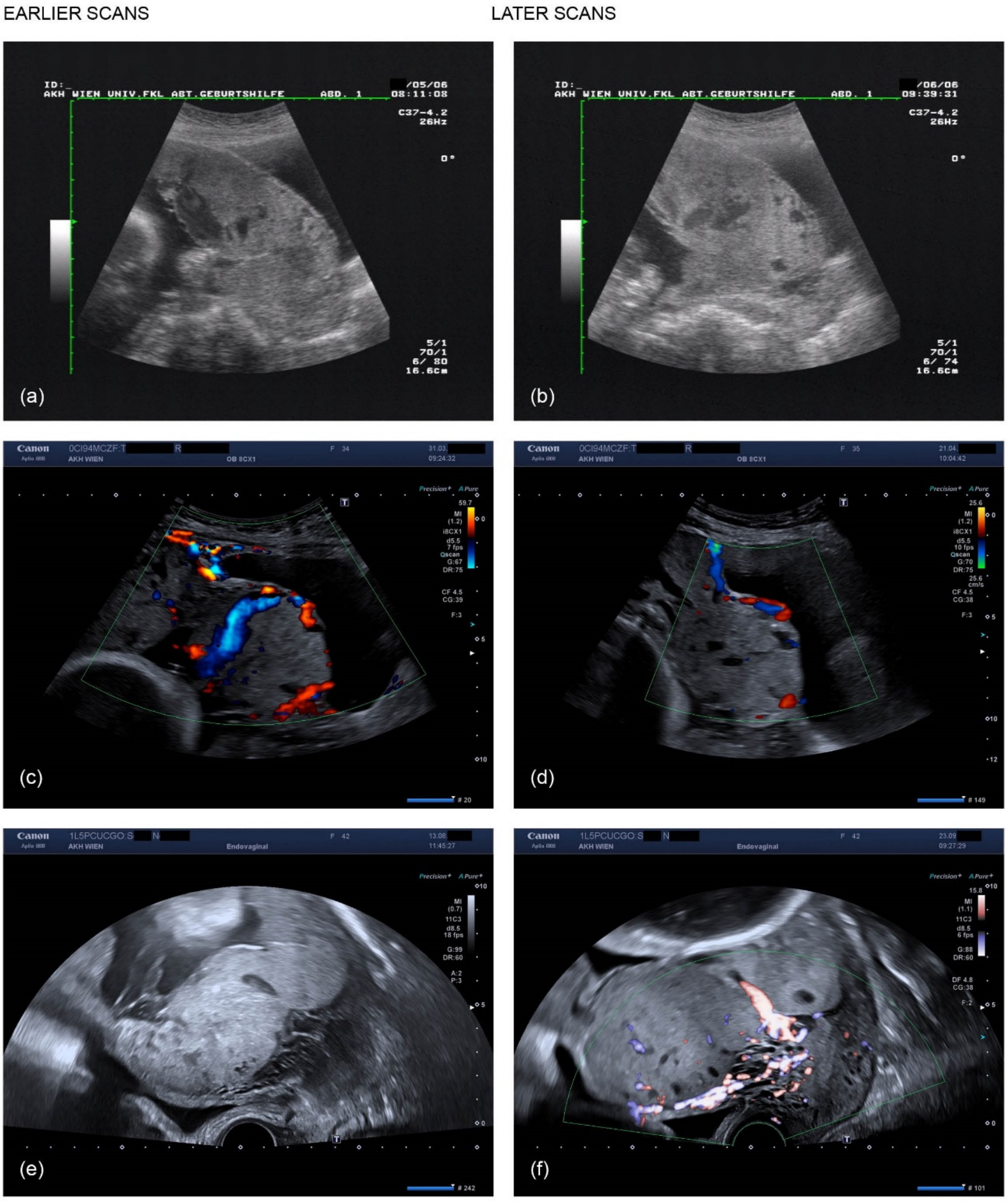

Figure S1. Example ultrasound pictures of 3 AIP cases, rows show the same patient, the earlier scans are depicted on the left, the later scans on the right; none of the later scans displays a diagnostic criterion for AIP, which is not visible on the earlier scans. (**a**,**b**) transabdominal (31 days between scans): visible myometrial thinning with partially absent myometrium + placental lacunae; (**c**,**d**) transabdominal (22 days between scans): pronounced placental bulge; (**e**,**f**) transvaginal (42 days between sans): precervically only serosa delineable, although this layer is also partially absent or interrupted by vascular convolutes indicating subplacental hypervascularity.
